# MEsenchymal StEm cells for Multiple Sclerosis (MESEMS): a randomized, double blind, cross-over phase I/II clinical trial with autologous mesenchymal stem cells for the therapy of multiple sclerosis

**DOI:** 10.1186/s13063-019-3346-z

**Published:** 2019-05-09

**Authors:** Antonio Uccelli, Alice Laroni, Lou Brundin, Michel Clanet, Oscar Fernandez, Seyed Massood Nabavi, Paolo A. Muraro, Roberto S. Oliveri, Ernst W. Radue, Johann Sellner, Per Soelberg Sorensen, Maria Pia Sormani, Jens Thomas Wuerfel, Mario A. Battaglia, Mark S. Freedman, on behalf of the MESEMS study group, Alice Laroni, Antonio Uccelli, Bruno Bonetti, Carolina Rush, Concepción Herrera, Cristina Ramo Tello, David Miller, David Szwajcer, Dirk Strunk, Donna Wall, Eduardo Aguera-Morales, Ernst W. Radue, Eva Rohde, Francesco Dazzi, Giancarlo Comi, Gianvito Martino, Guillermo Izquierdo Ayuso, H. Rabinovitch, Heather MacLean, James Marriott, Jens Thomas Wuerfel, Johann Sellner, Juan Racosta, Leila Arab, Lou Brundin, Maria Pia Sormani, Mario A. Battaglia, Mario Gimona, Mark S. Freedman, Martino Introna, Michel Clanet, Morten Blinkenberg, Naser Aghdami, Óscar Fernández, Paolo A. Muraro, Per Soelberg Sorensen, Rehiana Ali, Reza Vosoughi, Richard Nicholas, Roberto S. Oliveri, Ruth Ann Marrie, Seyed Massood Nabavi, Shahedeh Karimi

**Affiliations:** 1 Department of Neuroscience, Rehabilitation, Ophthalmology, Genetics, Maternal and Child Health and Center of Excellence for Biomedical Research (CEBR), University of Genova, Largo Daneo 3, 16132 Genoa, Italy; 2 IRCCS Ospedale Policlinico San Martino, Genoa, Italy; 3 Karolinska Institutet, R3:04 Karolinska University Hospital 171 76, Stockholm, Sweden; 4 CHU Toulouse, Université Paul Sabatier, INSERM UMR, 1043 Toulouse, France; 5 Instituto de Investigación Biomédica de Málaga (IBIMA), Regional University Hospital of Malaga, Malaga, Spain; 6 Department of Brain and Cognitive Sciences, Royan Institute for Stem Cell Biology and Technology, Royan, Iran; 7 ACCR, Iran and Regenerative Biomedicine Center, MS, Neurology Clinic and Research Unit, Tehran, Iran; 8 Department of Brain Sciences, Faculty of Medicine, Imperial College London, London, UK; 9 Cell Therapy Unit, Department of Clinical Immunology, Copenhagen University Hospital Rigshospitalet, Copenhagen, Denmark; 10 Medical Image Analysis Centre Basel (MIAC AG), Basel, Switzerland; 11 Department of Neurology, Christian Doppler Medical Center, Paracelsus Medical University, Salzburg, Austria; 12 Danish MS Center Department of Neurology, University of Copenhagen and Rigshospitalet, Copenhagen, Denmark; 13 Department of Health Sciences, University of Genoa, Genoa, Italy; 14 Department of Biomedical Engineering, University Basel, Basel, Switzerland; 15 Italian Multiple Sclerosis Foundation, Genoa, Italy; 16 Department of Life Sciences, University of Siena, Siena, Italy; 17 Department of Medicine (Neurology), University of Ottawa and the Ottawa Hospital Research Institute, Ottawa, Ontario Canada

**Keywords:** Multiple sclerosis, Mesenchymal stem cells, Mesenchymal stromal cells, Clinical trial

## Abstract

**Background:**

Multiple sclerosis (MS) is an inflammatory disease of the central nervous system with a degenerative component, leading to irreversible disability. Mesenchymal stem cells (MSC) have been shown to prevent inflammation and neurodegeneration in animal models of MS, but no large phase II clinical trials have yet assessed the exploratory efficacy of MSC for MS.

**Methods/design:**

This is an academic, investigator-initiated, randomized, double-blind, placebo-compared phase I/II clinical trial with autologous, bone-marrow derived MSC in MS. Enrolled subjects will receive autologous MSC at either baseline or at week 24, through a cross-over design. Primary co-objectives are to test safety and efficacy of MSC treatment compared to placebo at 6 months. Secondary objectives will evaluate the efficacy of MSC at clinical and MRI levels. In order to overcome funding constraints, the MEsenchymal StEm cells for Multiple Sclerosis (MESEMS) study has been designed to merge partially independent clinical trials, following harmonized protocols and sharing some key centralized procedures, including data collection and analyses.

**Discussion:**

Results will provide patients and the scientific community with data on the safety and efficacy of MSC for MS. The innovative approach utilized to obtain funds to support the MESEMS trial could represent a new model to circumvent limitation of funds encountered by academic trials.

**Trial registration:**

Andalusia: NCT01745783, registered on Dec 10, 2012.

Badalona: NCT02035514 EudraCT, 2010–024081–21. Registered on 2012.

Canada: ClinicalTrials.gov, NCT02239393. Registered on September 12, 2014.

Copenhagen: EudraCT, 2012–000518-13. Registered on June 21, 2012.

Italy: EudraCT, 2011–001295-19, and ClinicalTrials.gov, NCT01854957. Retrospectively registered on May 16, 2013.

London: Eudra CT 2012–002357-35, and ClinicalTrials.gov, NCT01606215. Registered on May 25, 2012.

Salzburg: EudraCT, 2015–000137-78. Registered on September 15, 2015.

Stockholm: ClinicalTrials.gov, NCT01730547. Registered on November 21, 2012.

Toulouse: ClinicalTrials.gov, NCT02403947. Registered on March 31, 2015.

**Electronic supplementary material:**

The online version of this article (10.1186/s13063-019-3346-z) contains supplementary material, which is available to authorized users.

## Background

The care of subjects with multiple sclerosis (MS) has been revolutionized over the past 30 years, from no treatment available until 1993, when interferon-beta 1b was approved in the US, to 13 disease-modifying drugs (DMD) approved in 2018 [1]. However, the evidence that the vast majority of DMD only affect relapsing MS makes the treatment scenario still unsatisfactory. MS is an inflammatory disease of the central nervous system (CNS) of autoimmune pathogenesis, characterized by demyelination and axonal loss. Most patients first experience a relapsing disease course characterized by episodes of neurologic disability followed by complete or partial recovery, which is believed to be driven by transient demyelination due to attack of immune cells coming into the CNS from the periphery (relapsing-remitting MS (RRMS)). RRMS is usually followed by a secondary progressive phase whose clinical hallmark is a progressive worsening of neurological status and establishment of irreversible disability (secondary progressive phase of disease (SPMS)). In about one out of five patients, the clinical course is progressive from the onset (primary progressive MS (PPMS)), though at least a quarter of such patients continue to show evidence of inflammation by way of clinical attacks and newly forming MRI lesions [2]. Immune mechanisms mainly driven by cells of the innate immune system and compartmentalized responses within the CNS together with non-immune mechanisms involving the CNS tissue, poorly targeted by available DMDs, are believed to contribute to the progressive phase [3, 4]. Prevention of cumulative disability and protection of the nervous tissue from the detrimental effects of inflammation are, therefore, the main goals of MS treatment research and particularly of that on stem cell-based treatments for MS.

Mesenchymal stem cells (MSC) are stromal [precursor] cells residing in many tissues, including the bone marrow (BM), where they support hematopoiesis. Treatment with MSC improves the course of the preclinical model of MS, experimental autoimmune encephalomyelitis (EAE), when administered at early stages. In EAE, MSC have a profound anti-inflammatory and immune-modulating effect [5–9], but they also exhibit neuroprotective features and foster remyelination endogenous neurogenesis with scarce evidence of differentiation in neural cells [6, 10, 11].

In humans, MSC, either autologous or allogeneic, have been used for more than 15 years in the treatment of graft-versus-host disease, as well as for other indications, showing a favorable safety profile [12, 13]. From these considerations, the first trials with BM-derived MSC for the treatment of MS were performed and published starting from 2007 [14–18]. These trials involved a limited number of subjects, with moderate to severe disability and active or progressive disease, treated with autologous, BM-derived MSC administered either by lumbar puncture (intrathecally) or intravenously (IV) and at variable dosages, as reviewed in [19]. Due to the low number of treated subjects, most trials were underpowered for drawing conclusions on efficacy and reported only data about the safety profile, which was overall favorable; one study assessed efficacy in ten patients with SPMS and visual impairment, finding that MSC treatment could improve some visual parameters compared to the pre-treatment [17]. An exploratory study with adipose tissue-derived MSC confirmed the safety of the treatment [20].

Here we present the protocol of the MEsenchymal StEm cells for Multiple Sclerosis (MESEMS) study, an academic, phase I/II clinical trial aimed at assessing safety as well as efficacy of a single intravenous (IV) dose of autologous BM-derived MSC for MS.

## Methods/design

### The MESEMS trial

The general protocol of the trial was devised by a panel of experts in stem cell research and/or in clinical research in MS, and was based on a consensus published in 2010 [21]. The MESEMS trial is aimed to assess the safety and efficacy of autologous, BM-derived MSC in a large cohort of subjects with active MS through a double-blind, randomized, cross-over design. A dose of 1–2 × 10^6^ MSC/kg body weight and the IV route were chosen, as reviewed in [22]. Given the data from preclinical experience showing that efficacy of MSC is achieved when cells are administered before the chronic phase of disease is reached, patients with relatively short disease duration (2 to 15 years since onset) will be enrolled. Patients may be enrolled with every form of disease (RRMS, SPMS, or PPMS), as long as there is evidence the disease is active, as defined by inclusion criteria specified below.

### The MESEMS network

The major constraint to perform an academic investigator-initiated phase 2 trial such as the MESEMS trial is funding. Additionally, autologous cells are legally classified in Europe as advanced therapy medicinal products (ATMPs) produced by good manufacturing practice (GMP)-authorized cell factories governed by regulation 1394/2007/EC. For ATMPs, clinical studies are regulated by each state through national guidelines requiring manufacturing authorization, resulting in regulatory and procedural consequences, which makes harmonization difficult.

To overcome these issues, we followed a novel strategy—a merging of partially independent clinical trials, following the same protocol and sharing some key centralized procedures, including data collection and analyses. Nine countries adhere to the MESEMS network, based in Italy (Genova, Milano, and Verona), Canada (Ottawa and Winnipeg), Austria (Salzburg), Denmark (Copenhagen), France (Toulouse), Iran (Tehran), Spain-Andalusia (Cordoba, Malaga, and Sevilla), Spain-Catalonia (Badalona), Sweden (Stockholm), and the United Kingdom (London). Centralized MRI reading and analyses were performed at the Medical Image Analysis Center (MIAC AG) in Basel, Switzerland, an imaging Contract Research Organization (CRO) specialized in phase II/III studies.

Trials performed within the MESEMS network share objectives and trial design, a central randomization procedure through a web-portal, central collection of data through a unique CRO based in Genova, Italy, and common funding of some centralized procedures by non-profit organizations. The trials have obtained independent authorization by national authorities (including amendments), have been registered to trial databases independently (either EudraCT or Clinicaltrials.gov, or both), employ MSC isolated and expanded by local cell factories, and may slightly differ in the patient population, as detailed below.

A Memorandum of Understanding was signed by all participating centers to guarantee compliance with the trial rules and sharing of data for centralized analysis. While the primary and secondary objectives are shared among the single trials of the MESEMS network and are going to be the object of a single publication, freedom is given to all centers to perform independent, ancillary studies to be published separately.

### Financial support

Non-profit organizations and academic entities funded the trial. Each national study was funded by national MS patients’ organizations and other non-profit agencies. Centralized activities were supported mainly by the Fondazione Italiana Sclerosi Multipla (FISM) through a grant to support the activities of the CRO and some of the centralized analysis of the MRIs at MIAC. Centralized activities were also supported by grants of the European Committee for Research in Multiple Sclerosis (ECTRIMS) and the Multiple Sclerosis International Foundation (MSIF).

### MESEMS trial design

MESEMS is a double-blind, randomized, cross-over, placebo-controlled trial lasting 56 weeks. The primary endpoints are safety (measured as number and severity of adverse events (AE)) of IV treatment with autologous BM-derived MSC and efficacy on brain MRI within the first 24 weeks after treatment. In detail, efficacy is defined as the reduction in the number of contrast(gadolinium-)-enhancing lesions (GEL) at 24 weeks in patients who received MSC at week 0 compared to those who received placebo. Secondary endpoints include efficacy at 48 weeks, comparison of early vs delayed treatment, and clinical efficacy at 24 and 48 weeks (Tables 1 and 2).

**Table 1:** Primary objectives of the MESEMS project

Aim	Measures
Safety of IV therapy with autologous BM-derived MSC in RRMS, SPMS, and PPMS subjects	Number and severity of AEs within each treatment arm
Activity of autologous BM-derived MSCs in MS subjects	Reduction compared to placebo in the total number of contrast-enhancing lesions (GEL) at MRI acquired on conventional MRI scans (minimum magnetic field intensity 1.5 T) over 24 weeks

**Table 2:** Secondary objectives of the MESEMS project

Aim	Measures
To compare the number of active MRI lesions in the placebo vs active treatment periods in both groups	Number of GEL counted over weeks 28, 36, and 48 (cross-over re-treatment) compared with the number of GEL counted over 4, 12, and 24 weeks (placebo vs active treatment periods) within each group
To evaluate efficacy of MSC in reducing combined MRI activity and volume of black holes (BH) in both treatment groups at 24 weeks	Combined unique MRI activity (number of new or enlarging T2w, or enhancing or re-enhancing lesions) and volume of GEL over 4, 12, and 24 weeks compared between treatment groups. Volume of BH over 24 weeks compared between treatment groups
To compare combined MRI activity and volume of BH in the placebo vs active treatment periods in both groups	Combined unique MRI activity and volume of GEL over weeks 28, 36, and 48 (cross-over re-treatment) compared with the same outcomes over 4, 12, and 24 weeks (placebo vs active treatment periods) within each group. Volume of BH over week 48 (cross-over re-treatment) compared with the same outcome over week 24 week (placebo vs active treatment periods) within each group
To evaluate efficacy of MSC in reducing the volume of T2 lesions in both treatment groups at 24 weeks	Volume of T2w lesions over 24 weeks compared between treatment groups
To compare the volume of T2 lesions in the placebo vs active treatment periods in both groups	Volume of T2w lesions over week 48 (cross-over re-treatment) compared with the same outcome over week 24 week (placebo vs active treatment periods) within each group
To evaluate efficacy of MSC in reducing relapses at 24 weeks and to compare the number of relapses in the placebo vs active treatment periods in both groups	Number of relapses in MSC treatment group vs placebo group in the first 24 weeks and after cross-over re-treatment in the two groups (see below for definition of relapse)
To evaluate efficacy of MSC in reducing the time to sustained progression of disability and increasing the number of progression-free patients at 24 weeks and to compare the time to sustained progression of disability and the proportion of progression-free patients in the placebo vs active treatment periods in both groups*	Time to sustained progression of disability and proportion of progression-free patients compared between treatment groups during the first 24 weeks and after cross-over re-treatment in the two groups
To evaluate efficacy of MSC in increasing the number of progression-free patients at 24 weeks and to compare the proportion of disease-free patients in the placebo vs active treatment periods in both groups§	Proportion of disease-free patients compared between treatment groups during the first 24 weeks and after cross-over re-treatment in the two groups
To evaluate the efficacy of MSC treatment in clinical scores such a Multiple Sclerosis Functional Composite (MSFC) score and Symbol Digit Modalities Test (SDMT) score	Changes in MSFC and SDMT scores in the MSC treated group vs placebo group during the first 24 weeks and after cross-over re-treatment in the two groups

*Sustained progression of disease is defined as any 6-month sustained increase in EDSS: for baseline EDSS < 5.5, any 1-point EDSS increase; for baseline EDSS ≥ 5.5, any 0.5-point EDSS increase

^§^Disease-free: patients without relapses, with no evidence of sustained progression of disability and new MRI activity

**Fig. 1:**
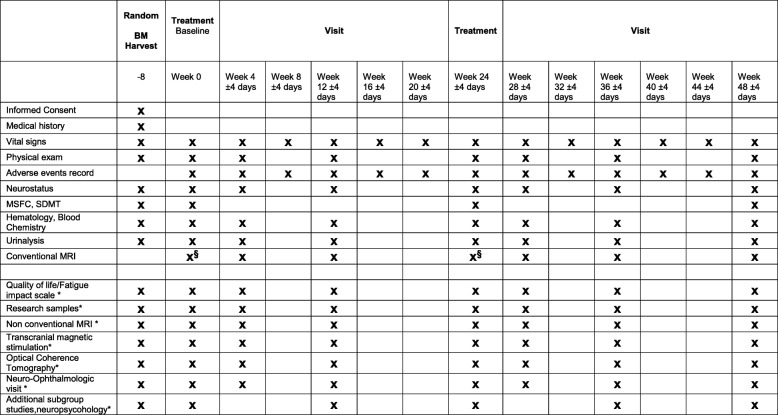
Schedule of enrolment, interventions, and assessments of the MESEMS trial. *Optional studies as per sites desire; ^§^MRI at week 0 and week 24 must be performed before the IV treatment with MSC or placebo

**Fig. 2:**
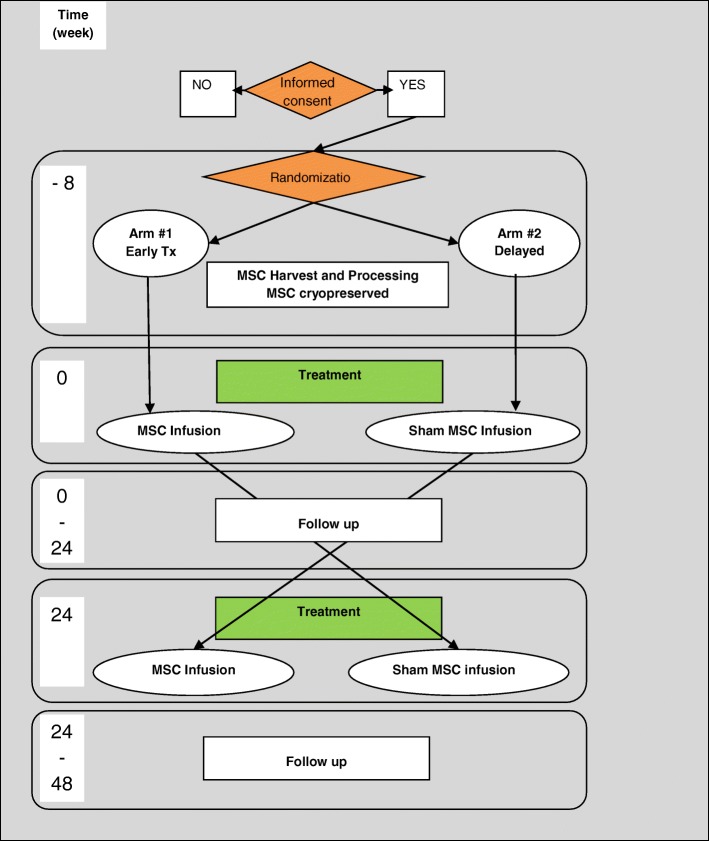
Study design and patient flow in the MESEMS trial

The schedule of assessment is reported in Fig. 1 and a schematic representation of the trial is depicted in Fig. 2. Patients enrolled in trials belonging to the MESEMS network, after informed consent, undergo central randomization and a BM aspirate (at week − 8). Cell factories GMP-approved by national authorities expand the BM-derived MSC up to 1–2 × 10^6^/kg of subject body weight and, after the appropriate quality checks, freeze one IV bag of MSC in infusion medium and another IV bag containing infusion medium (including cryoprotectant) only with no cells (i.e., placebo). According to the central randomization code, each patient receives one bag at baseline (week 0) and the other at week 24. Patients and investigators are blinded to the treatment. For evaluation of safety, AE occurrence and severity are registered monthly since inclusion according to the CTC-AE classification. For evaluation of efficacy, patients undergo a contrast-enhanced brain MRI at baseline, followed by six further MRI visits.

### Inclusion criteria

Patients included in the MESEMS trials must be aged 18–50 years, have short disease duration (2–15 years since MS onset, included), and moderate to severe disability (Expanded Disability Status Scale 2.5 to 6.5, included). All trials belonging to the MESEMS network allow inclusion of MS patients with the relapsing-remitting (RR) form of disease, with recent relapse(s) and/or MRI activity, as detailed in Table 3. Inclusion criteria for RRMS were modified in the second version of the protocol in order to define recent MRI activity not only as detection of one GEL at MRI performed within the last 12 months, but also as detection of a new T2 lesion at MRI performed within the last 12 months compared to a previous MRI performed within the last 12 months. Most trials with one exception allow for the inclusion of patients with the progressive form of disease. For the secondary progressive (SP) form of disease, progression of disease in the past year, in the presence of relapses or MRI activity, are required (Table 3), and for the primary progressive (PP) form, progression of disease and MRI activity in the past year are mandatory for inclusion (Table 3). Very recent MRI activity (within the last 3 months) is an additional requirement for patients affected by all disease forms and enrolled in the trial based in London in order to capture highly active MS individuals.

**Table 3:** Inclusion criteria of the MESEMS trial

	Italy	Spain–Andalusia	Copenhagen	London	Stockholm	Toulouse	Spain–Catalonia	Salzburg	Tehran
RRMS with:									
≥ 1 clinically documented relapse in past 12 months	x	x	See below	See below	x	x	x	x	
≥ 2 clinically documented relapses in last 24 months	x	x			x	x	x	x	x
≥ 1 GEL at MRI performed within the last 12 months or new T2 lesion at MRI performed within the last 12 months compared to a previous MRI performed within the last 12 months.	x	x			x	x	x	x	x
		London: ≥ 1 moderate-severe relapse AND (1 or more GELs in past 18 months OR ≥ 1 new T2 lesion). PLUS: ≥ 1 on MRI within 3 months prior to harvesting							
		Copenhagen ≥ 1 moderate-severe relapse in past 18 months OR ≥ 1 GEL (double-triple Gad) OR ≥ 1 new T2							
SPMS with:									
An increase of ≥ 1 EDSS point (if at randomization EDSS ≤ 5.0) or 0.5 EDSS point (if at randomization EDSS ≥ 5.5) in the last 12 months AND ≥ 1 clinically documented relapse or ≥ 1 GEL at MRI within the last 12 months	x	x	No SPMS	See below	x	x	x	x	x
Other (specify)		London: Changes in EDSS relate to previous 18 months AND 1(+) GELs in last 18 months or require SPMS with 1(+) relapses and 1(+) T2 lesions in past 18 months							
PPMS with:									
An increase of ≥ 1 EDSS point (if at randomization EDSS ≤ 5.0) or 0.5 EDSS point (if at randomization EDSS ≥ 5.5), in the last 12 months	x	x	No PPMS	See below	x	x	x	x	x
AND ≥ 1 GEL at MRI performed within the last 12 months	x	x			x	x	x	x	x
AND Positive cerebrospinal fluid (CSF) (oligoclonal banding)	x	x			x	x	x	x	x
		London: EDSS changes, GELs, or T2 lesions over last 18 months. PLUS: ≥ 1 on MRI within 3 months prior to harvesting							

Shared exclusion criteria are: disease form not fulfilling inclusion criteria; any active or chronic infection, including infection with HIV1/2 or chronic hepatitis B or hepatitis C; any chronic or acute disease that could be not compatible with the trial protocol as per clinician’s judgment; treatment with any immunosuppressive therapy, including natalizumab and fingolimod, within the 3 months prior to randomization; treatment with interferon-beta or glatiramer acetate within the 30 days prior to randomization; treatment with alemtuzumab within the 12 months prior to randomization; treatment with rituximab/other anti-CD20 drug within the 6 months prior to randomization; treatment with corticosteroids within the 30 days prior to randomization; relapse occurring during the 60 days prior to randomization (except for the trial based in London); previous history of a malignancy other than basal cell carcinoma of the skin or carcinoma in situ that has been in remission for more than one year (except for the trial based in Copenhagen); severely limited life expectancy by another co-morbid illness; history of previous diagnosis of myelodysplasia or previous hematologic disease or current clinically relevant abnormalities of white blood cell counts; pregnancy or risk of pregnancy (this includes patients that are unwilling to practice active contraception during the duration of the study); estimated glomerular filtration rate < 60 mL/min/1.73 m^2^ or known renal failure or inability to undergo MRI examination; inability to give written informed consent in accordance with research ethics board guidelines.

### MSC preparation and culture

Upon arrival of BM at the cell factory within 12 h after harvesting, MSC are expanded up to 1–2 × 10^6^/kg of body weight of the patient and frozen until usage; the Andalusian cell factory follows a different procedure as it freezes the BM cells upon arrival and expands the MSC just before infusion without further freezing. Cell culture media include alpha-minimum essential medium (alpha-MEM) and low-glucose Dulbecco’s modified Eagle medium (D-MEM). The growth supplement is human platelet lysate or fetal bovine serum (depending on the protocol adopted by each cell factory). Cells are grown in incubators at ambient oxygen level (21%). MSC are harvested after a maximum number of two passages. The final product is tested to exclude growth of aerobic and anaerobic bacteria and *Mycoplasma* and defined as adherent cells positive for CD73, CD90, and CD105 and negative for CD14, CD34, and CD45, as described later. Moreover, cytogenetic analysis is performed to rule out an abnormal karyotype, as described below.

### Characteristics of the final MSC product

MSCs may be released from the laboratory for clinical use if they fulfill the following criteria:

- CD105 expression ≥ 70%
- CD73 expression ≥ 70%
- CD90 expression ≥ 70%
- CD14 expression ≤ 10%
- CD45 expression ≤ 10%
- CD34 expression ≤ 10%
- Viability ≥ 80% at the time of freezing and in an aliquot thawed 2–3 weeks after freezing
- Detection of ≥ 70% expression of CD73, CD90, and CD105 in an aliquot thawed 2–3 weeks after freezing
- Lack of colony forming capacity in methylcellulose
- Lack of abnormal karyotype detected in at least 20 metaphases analyzed (unless an insufficient number of metaphases is obtained due to low cell growth as evidenced by the mitotic index)

### Statistical analysis

#### Sample size

To evaluate the safety of MSC infusion, the number, timeframe of occurrence, and severity of AE will be recorded in MSC and placebo treatment groups. For the evaluation of efficacy, a sample size of 160 patients was estimated to give 80% power at a significance level of 5% to detect a decrease of 50% in the number of GEL counted during the 6-month duration of treatment. This number was estimated assuming that the average number of total GEL on three MRI scans (performed at weeks 4, 12, and 24) will be 7.4 (SD = 11.4), that is, half of the total number of GEL counted over six monthly MRIs in the phase II study of the oral drug fingolimod for MS [23]. Given a possible drop-out rate of 15% (*N* = 24), the total number of patients to enroll will be 185.

#### Data analysis

For the primary outcome, comparisons of the incidence and severity of adverse events between treatment groups will be carried out by the chi-square test.

For the co-primary outcome, the total number of GEL lesions at MRI at weeks 4, 12, and 24 will be compared between treatment groups using a negative binomial (NB) regression analysis, adjusting for the number of GEL at baseline.

For secondary outcomes, the total number of GEL at MRI at weeks 4, 12, and 24 in each treatment group will be compared with the number of GEL lesions at MRI at weeks 28, 36, and 48 following the infusion using a non-parametric statistic for paired data. The dependence on time of the total number of GEL at MRI will be analyzed in the group originally randomized to active treatment using a mixed effect generalized model with NB errors. The total number of new or enlarging T2w lesions at weeks 4, 12, and 24 post-treatment will be contrasted in the two groups using a NB regression analysis with treatment group and baseline number of GEL lesions as covariate. MRI lesion volumes will be log-transformed and compared by ANOVA models between groups. The total number of combined unique lesions, at week 4, 12 and 24 weeks post-treatment will be contrasted in the two groups using a NB regression analysis with treatment group and baseline number of GEL lesions as covariate. MRI lesion volumes will be log-transformed and compared by ANOVA models between groups. The combined unique MRI activity, number of new T2w lesions, volume of GEL over weeks 28, 36, and 48, the T2w and the BH volume changes between weeks 24 and 48 (cross-over re-treatment) will be compared with combined unique MRI activity, number of new T2w lesions, volume of GEL over 4, 12, and 24 weeks, T2w and BH volume changes between baseline and week 24 (placebo vs active treatment periods) using a non-parametric statistic for paired data. The number of relapses in the MSC treatment group vs the placebo group will be compared using a NB regression analysis. Time to sustained progression of disability (RRMS group) will be analyzed with a Cox proportional-hazards model. The proportion of progression-free patients in the two groups will be compared by using the chi-square test (RRMS patients). Mean changes in Multiple Sclerosis Functional Composite (MSFC) score and in SDMT score in MSC treatment group vs placebo group compared to baseline will be analyzed with a one-way ANOVA test.

#### Assessment of inter-center differences

Stringent phenotypic criteria have been adopted for MSC cell release. Moreover, we adopted an electronic case record form (eCRF) containing very detailed templates related to MSC production. However, the primary analysis will not be adjusted for center effect due to the small number of patients expected to be enrolled per center. As such, no subgroup analysis has been planned per study protocol. Regarding the possible variability in the number of infused cells, the stem cell relevant templates in the eCRF contain information on MSC viability in the final cell product; therefore, an exploratory analysis will be run to correlate the total number of infused MSCs—a continuous variable allowing such type of analysis—to efficacy outcome. However, due to the small expected sample size, this analysis will not be powered to detect significant treatment effect differences and will only be used to generate hypotheses.

### Treatment of relapses

Any patient complaining of a new neurological symptom suggestive of relapse should be referred to the examining neurologist for EDSS assessment. Investigators may treat relapses with intravenous methylprendnisolone, 1000 mg, for 3 to 5 consecutive days.

### Prohibited concomitant medications

The treatment with immunmodulatory or immunosuppressive drugs for MS during the trial is prohibited.

### Centralized study procedures

#### Centralized randomization and data entry

Once all the eligibility criteria have been checked, and patients have signed the informed consent, they will be randomized through the randomization website (https://trials.actide.com/mesems/live/public/en/users/login) administered by the CRO “Latis”, which provides the investigators with access credentials.

Throughout the study, investigators are required to fill an eCRF, administered by the CRO and accessible with personal credential from the website https://trials.actide.com/mesems/live/public/en/users/login.

#### Centralized reading of the MRI

Scanners for MRI must have a minimum magnetic field intensity of 1.5 T. In order to ensure optimal MRI data quality, a “dummy run” scan must be uploaded by each center through the MIAC web-portal and, upon revision, quality must be accepted before patient’s enrollment. Patients undergo seven MRI visits at defined timepoints, as detailed before and in the schedule of assessment; MRI scans scheduled for week 0 and week 24 must be performed before the treatment. MRI data are uploaded electronically to the MIAC website and analyzed centrally in a blinded fashion.

### On-site procedures

#### Bone marrow harvest

BM will be harvested at randomization visit according to Good Clinical Practice guidelines. Briefly, a total of 10 to 100 ml of BM aspirate will be collected after performing multiple small incisions and perforations from the posterior-superior iliac spine under local anesthesia. Anticoagulant will be added to the aspiration syringes and to the bag where the bone marrow will be collected prior to delivery to the cell factory under computer-controlled temperature (not at all sites).

#### Administration of the investigational product

At week 0 a single infusion of either ex vivo expanded autologous MSC or placebo will be administered IV, according to randomization, after premedication with normal saline and an anti-histamine drug to prevent infusion reactions to AB plasma or to dimethyl sulfoxide, which are in the MSC bag as well as the placebo bag (concomitant treatment with steroids is not allowed at time of infusion). Subjects will be monitored for up to 2 h following administration. At week 24, another infusion will be performed for cross-over re-treatment: at week 24, treatments will be reversed compared to week 0 (i.e., patients who received initial MSCs will receive suspension media and vice versa) and will be administered following the same protocol as described for week 0.

#### Follow-up visits

At scheduled visits, vital signs and AE will be recorded. Other procedures will be performed according to the study schedule as depicted in Table 1. Investigators will be divided into “treating neurologist”, the physician responsible for the patient care, AE recording and treatment administration, and “examining neurologist”, the physician performing the neurological evaluation.

SPIRIT checkist for this trial is reported in Additional file 1.

## Monitoring of AE

### All AE

All AEs that occur between the first study-related procedure and 6 months post-administration of the last dose of study drug (or after this date if the investigator feels the event is related to the study drug) must be recorded by investigators in the eCRF. Those meeting the definition of a serious adverse event (SAE) related or not to the study treatment must be reported within 24 h to the CRO.

### Severity

Severity for each AE, including any laboratory abnormality, will be determined by using the National Cancer Institute (NCI) Common Terminology Criteria for Adverse Events (CTCAE, version 4.0) as a guideline, wherever possible. The criteria are available online at  https://ctep.cancer.gov/protocoldevelopment/electronic_applications/ctc.htm#ctc_40.

In those cases where the CTCAE criteria do not apply, severity should be defined according to the following criteria:

- Mild (grade 1): Awareness of signs or symptoms, but easily tolerated
- Moderate (grade 2): Discomfort enough to cause interference with normal daily activities
- Severe (grade 3): Inability to perform normal daily activities
- Life threatening (grade 4): Immediate risk of death from the reaction as it occurred

### Causality

Relationship to study drug administration will be determined as follows:

- *None:* No relationship between the experience and the administration of the study drug; related to other etiologies such as concomitant medications or patient’s clinical state.
- *Unlikely:* The current state of knowledge indicates that the relationship is unlikely.
- *Possible:* A reaction that follows a plausible temporal sequence from administration of the study drug and follows a known response pattern to the suspected study drug. The reaction might also have been produced by the patient’s clinical state or other modes of therapy administered to the patient.
- *Probable:* A reaction that follows a plausible temporal sequence from administration of the study drug and follows a known response pattern to the suspected study drug. The reaction cannot be reasonably explained by the known characteristics of the patient’s clinical state or other modes of therapy administered to the patient.
- *Definite:* An AE which is listed as a possible adverse reaction and cannot be reasonably explained by an alternative explanation, e.g., concomitant drug(s), concomitant disease(s).

### Serious adverse events

The enrolling sites will be responsible for pharmacovigilance. Events defined as serious must be reported by the centers according to Good Clinical Practice and the local protocol.

The following attributes must be assigned when reporting:

- Detailed description of the event
- Date of onset and date of resolution
- Severity of the event (see “Severity” section for details)
- Assessment of relatedness to study drugs (see “Causality” section for definitions) and action taken
- Other suspect drugs/devices
- Outcome

All SAEs will be followed up until resolution or study end. The investigator will be asked to provide interim and follow-up reports, as necessary, if the SAE has not resolved at the time of initial report. All deaths (CTCAE grade 5) occurring during the study must be reported as an SAE.

### Monitoring

Patient registration, randomization and data entry of all clinical, laboratory, and MRI data will be performed via an eCRF accessible through the web by authorized researchers and administered by the CRO. The principal investigator of every trial will be responsible for ensuring that medical and paramedical staff are allocated to provide adequate source documentation and to fill the required items in the eCRF. The eCRF should be submitted within 2 weeks of completion of the study treatment and each study follow-up visit. All study-related eCRFs will be stored for 5 years in the archives of each center.

Study management, monitoring, and database management will be the responsibility of the CRO–Latis (Genova, Italy), whereas design and biostatistical analysis will be guaranteed by the Biostatistics Unit, Department of Health Sciences of the University of Genoa (Italy).

An International Trial Steering Committee (ISC) was appointed to provide guidance to the trial. Members of the ISC are listed in Additional file 2. Furthermore, four independent researchers, experts in the field of adult stem cells, clinical trials in MS, neuroimmunology, and neuroradiology, respectively, who oversee monitoring of the safety of the treatment, compose the Independent Data Safety Monitoring Committee (IDSMC). Members of the IDSMC are listed in Additional file 2.

The ISC comprises the principal investigator of each national trial, as well as other expert representatives essential for the conduct of the international collaborative trial, including in statistics, MRI, and cell production. The ISC will be responsible for the actual conduct of the trial and will decide on the distribution of funds received from the international funding bodies. It will meet once or twice yearly to recapitulate the progress of the trial, discuss issues that may arise about study procedures, and make decisions based on the recommendations of the IDSMC. Decisions will be implemented upon majority agreement of members.

Members of the IDSMC have accepted to serve as members of the IDSMC under a confidentiality agreement. Through quarterly meetings, they will review safety and toxicity reports (tables and listings) they will receive directly from the CRO and give their recommendations thereof to ensure subjects’ safety throughout the trial. In addition, the IDSMC will be requested by the CRO to give their recommendations in individual cases where an unexpected SAE clearly related to the treatment has occurred. Unblinding is restricted to emergency situations. It should be used only under circumstances where the knowledge of the treatment is necessary for the proper handling of the patient. In this case a “Request of unblinding form” must be completed by the investigator and sent by fax/email to the CRO, which will communicate by fax/email the treatment to the Investigators in a timely manner.

### Patient confidentiality

In order to maintain patient privacy, all data capture records, study drug accountability records, and study reports and communications will identify the patient by initials and the assigned patient number. The investigator will grant the study monitor(s) and auditor(s) access to the patient’s original medical records for verification of data gathered on the data capture records and to audit the data collection process. The patient’s confidentiality will be maintained and will not be made publicly available to the extent permitted by the applicable laws and regulations.

### Premature closure of the study

This study may be prematurely terminated if, in the opinion of the investigator, there is sufficient reasonable cause. The terminating party will provide written notification documenting the reason for study termination to the investigator. Circumstances that may warrant termination include, but are not limited to:

- Treatment-related mortality: in case of one fatal accident 24 h after treatment, the IDSMC will evaluate the case and decide whether the trial should be stopped. The trial is stopped after the second fatal event 24 h after treatment.
- Determination of unexpected, significant, or unacceptable risk to patients.
- Failure to enter patients at an acceptable rate.
- Insufficient adherence to protocol requirements.
- Insufficient complete and/or valuable data.
- Protocol violation.
- Administrative decision.
- New data able to influence the willingness of the investigators to pursue the study.

### Use of information and publication

The results of the study will be reported in a Clinical Study Report generated by the sponsors and will contain all data from all investigational sites. Any work created in connection with performance of the study and contained in the data that can benefit from copyright protection (except any publication by the investigator as provided for below) shall be the property of the sponsor as author and owner of copyright in such work. The participating centers will recognize the integrity of a double center study by not publishing data derived from the individual site until the combined results from the completed study have been published in full. Authorship of publications resulting from this study will be based on generally accepted criteria for major medical journals.

### Discussion and potential impact

The MESEMS trial will provide patients and scientific communities with the answers to two key questions about MSC-based treatment of MS: 1) whether autologous, BM-derived MSC are safe in the MS population; and 2) whether autologous, BM-derived MSC are efficacious in decreasing MS activity.

Stem cell-based therapies are often advertised to the public as a panacea for every disease with a degenerative component, for their supposed capability to regenerate diseased tissues through engraftment paracrine effects and/or transdifferentiation, despite the lack of scientific evidence and clinical proof of concept (http://www.nature.com/news/italian-stem-cell-trial-based-on-flawed-data-1.13329) [24]. Preclinical evidence shows that MSC are indeed effective in an animal model of MS, but their mechanism of action appears to involve a combination of modulation of the peripheral immune system and promoting tissue protection. The small numbers of patients involved in the published trials with MSC for MS do not allow us to draw conclusions on whether preclinical findings are applicable to human beings.

Based on the sample size calculation, the MESEMS trial design should overcome such methodological issues by enrolling enough patients for analyzing efficacy on MRI metrics. MRI activity is a recognized marker for clinical activity (i.e., relapses) in MS and is the primary efficacy outcome in most phase II clinical trials with novel drugs for relapsing MS [25].

Importantly, the MESEMS trial is not powered to give conclusive answers on whether MSC are effective in promoting tissue protection, i.e., in preventing disability, but rather seeks as a phase 2 trial to assess exploratory efficacy. Accordingly, confirmatory efficacy results need to be obtained with a larger subject study population and a longer follow-up period, which is beyond the scope of this collaborative phase 2 effort. However, analysis of secondary endpoints will gather some preliminary information which could be possibly utilized to design a future, confirmatory phase III trial.

The trial has the peculiarity of being composed of several national trials, partially independent but sharing the protocol and some key procedures, such as central randomization and data collection. Such an innovative trial design, created in order to overcome financial and technical obstacles that would have otherwise prevented us from carrying out the study, has obtained support and endorsement by eminent charities and funding bodies, such as FISM, ECTRIMS, and the MSIF (“We appreciate the challenges involved in bringing together a number of smaller datasets and applying a common analysis plan to them but believe this to be an important step in getting the most from the data” – letter of endorsement to the study from Peer Baneke, Chief Executive and Alan Thompson, Chairman, IMSB of MSIF on February 23, 2012).

Such a collaborative group could constitute an example for other research groups aiming to assess the efficacy of stem cell-based treatments for other neurologic diseases such as, among others, Parkinson’s disease, amyotrophic lateral sclerosis, or spinal cord injury, where clinical trials with MSC have been performed so far in limited numbers of patients [19].

## Additional files

Additional file 1: SPIRIT 2013 checklist: Recommended items to address in a clinical trial protocol and related documents. (DOC 256 kb)

Additional file 2: Supplementary materials. (DOCX 27 kb)
