# Supplementary material for: MEsenchymal StEm cells for Multiple Sclerosis (MESEMS): a randomized, double blind, cross-over phase I/II clinical trial with autologous mesenchymal stem cells for the therapy of multiple sclerosis
Source: Trials. 2019 May 9;20:263. doi: 10.1186/s13063-019-3346-z (PMC6507027; doi:10.1186/s13063-019-3346-z)
Supplement: Supplementary file 2 — Supplementary materials. (DOCX 27 kb) [file 13063_2019_3346_MOESM2_ESM.docx]

Supplementary material

**The following individuals are members of the MESEMS study group:**

Antonio Uccelli (University of Genova and IRCCS Ospedale Policlinico San Martino, Genova, Italy); Alice Laroni (University of Genova and IRCCS Ospedale Policlinico San Martino, Genova, Italy)^;^ Martino Introna (Center of Cellular Therapy "G. Lanzani", Azienda Socio Sanitaria Territoriale Papa Giovanni XXIII, Bergamo, Italy); Gianvito Martino (Neuroimmunology Unit, Department of Neuroscience, Institute of Experimental Neurology, San Raffaele Scientific Institute, Milan, Italy); Bruno Bonetti (University of Verona, Verona, Italy, Giancarlo Comi (Department of Neurology and Institute of Experimental Neurology, Vita-Salute San Raffaele University, San Raffaele Hospital, Milan, Italy); Lou Brundin (Karolinska Institutet, R3:04 Karolinska University Hospital 171 76 Stockholm Sweden); Michel Clanet (CHU Toulouse, Université Paul Sabatier , INSERM UMR 1043, Toulouse France); Óscar Fernández (Institute of Biomedical Research (IBIMA). Regional University Hospital of Malaga. Malaga, Spain); Guillermo Izquierdo Ayuso (Hospital Vithas, Sevilla, Spain); Eduardo Aguera-Morales (Department of Neurology, Reina Sofía University Hospital. IMIBIC. University of Cordoba, Spain); Concepción Herrera (Department of Hematology and Cell Therapy Unit, Reina Sofía University Hospital. IMIBIC. University of Cordoba, Spain); Cristina Ramo Tello (Neuroscience Department, Hospital Germans Trias-i-Pujol, Badalona, Spain); Seyed Massood Nabavi(Regenerative Biomedicine center, Royan institute of stem cell biology and technology, Tehran, Iran); Naser Aghdami(Regenerative Biomedicine center, Royan institute of stem cell biology and technology, Tehran, Iran); Shahedeh Karimi(Regenerative Biomedicine center, Royan institute of stem cell biology and technology, Tehran, Iran); Leila Arab(Regenerative Biomedicine center, Royan institute of stem cell biology and technology, Tehran, Iran); Paolo A. Muraro (Department of Brain Sciences, Faculty of Medicine, Imperial College London, London, UK); Rehiana Ali, (Imperial College London, London UK); Richard Nicholas ( Imperial College Healthcare Trust, London, UK); David Miller (University College London, London UK); Francesco Dazzi (King’s College, London UK);

Roberto S. Oliveri (Cell Therapy Unit, Department of Clinical Immunology, Copenhagen University Hospital Rigshospitalet, Copenhagen, Denmark); Ernst W Radue (Medical Image Analysis Centre Basel (MIAC AG) Basel, Switzerland); Johann Sellner (Department of Neurology, Christian Doppler Medical Center, Paracelsus Medical University, Salzburg, Austria); Eva Rohde (Department of Blood Group Serology and Transfusion Medicine, Paracelsus Medical University Hospital Salzburg, Salzburg, Austria); Mario Gimona (GMP Unit, Spinal Cord Injury and Tissue Regeneration Center Salzburg (SCI-TReCS), Paracelsus Medical University, Salzburg, Austria); Dirk Strunk (Institute of Experimental and Clinical Cell Therapy, Paracelsus Medical University, Salzburg, Austria); Per Soelberg Sorensen (Danish MS Center Department of Neurology, University of Copenhagen and Rigshospitalet, Copenhagen, Denmark); Morten Blinkenberg (Danish Multiple Sclerosis Center, Department of Neurology, University of Copenhagen, Rigshospitalet, Copenhagen, Denmark); Maria Pia Sormani (Department of Health Sciences, University of Genoa, Genoa, Italy); Jens Thomas Wuerfel (Medical Image Analysis Centre Basel (MIAC AG) and Department of Biomedical Engineering, University Basel, Switzerland); Mario A. Battaglia^,^  (Italian Multiple Sclerosis Foundation, Genoa, Italy and Department of Life Sciences, University of Siena, Siena, Italy); Mark S. Freedman (Department of Medicine (Neurology), University of Ottawa and the Ottawa Hospital Research Institute, Ottawa, Ontario, Canada); Heather MacLean (University of Ottawa and the Ottawa Hospital Research Institute, Ottawa, Ontario, Canada); Carolina Rush (University of Ottawa and the Ottawa Hospital Research Institute, Ottawa, Ontario, Canada); H. Rabinovitch (University of Ottawa and the Ottawa Hospital Research Institute, Ottawa, Ontario, Canada); James Marriott (Max Rady College of Medicine, Rady Faculty of Health Sciences, University of Manitoba, Winnipeg, Manitoba); Ruth Ann Marrie(Max Rady College of Medicine, Rady Faculty of Health Sciences, University of Manitoba, Winnipeg, Manitoba); David Szwajcer(Max Rady College of Medicine, Rady Faculty of Health Sciences, University of Manitoba, Winnipeg, Manitoba); Donna Wall(Max Rady College of Medicine, Rady Faculty of Health Sciences, University of Manitoba, Winnipeg, Manitoba); Reza Vosoughi (Max Rady College of Medicine, Rady Faculty of Health Sciences, University of Manitoba, Winnipeg, Manitoba); Juan Racosta (Max Rady College of Medicine, Rady Faculty of Health Sciences, University of Manitoba, Winnipeg, Manitoba).

**Memebrs of the International Trial Steering Committee (ISC) of the MESEMS trial**

Antonio Uccelli(Co-Principal Investigator), University of Genova, IRCCS Ospedale Policlinico San Martino, Genova Italy

Mark S. Freedman,(Co-Principal Investigator),^,^  University of Ottawa and the Ottawa Hospital Research Institute, Ottawa, Ontario, Canada

Lou Brundin, Karolinska Institutet,. Stockholm Sweden

Michel Clanet, CHU Toulouse, Université Paul Sabatier , INSERM UMR 1043, Toulouse France

Oscar Fernandez, Instituto de Investigación Biomédica de Málaga (IBIMA). Regional University Hospital of Malaga, Malaga, SPAIN

Seyed Massood Nabavi, Royan institute for stem cell biology and technology, Royan, Iran, ACCR, Iran and Regenerative Biomedicine center, MS, Neurology clinic and research unit, Tehran, Iran

Paolo A. Muraro, Department of Brain Sciences, Faculty of Medicine, Imperial College London, London, UK

Roberto S. Oliveri, Cell Therapy Unit, Department of Clinical Immunology, Copenhagen University Hospital Rigshospitalet, Copenhagen, Denmark

Ernst W. Radue, Medical Image Analysis Centre Basel (MIAC AG) Basel, Switzerland

Johann Sellner, Department of Neurology, Christian Doppler Medical Center, Paracelsus Medical University, Salzburg, Austria

Per Soelberg Sorensen Danish MS Center Department of Neurology, University of Copenhagen and Rigshospitalet, Copenhagen, Denmark

Maria Pia Sormani, University of Genoa, Genoa, Italy

Jens Thomas Wuerfel , Medical Image Analysis Centre Basel (MIAC AG), University Basel, Switzerland

**Members of the IDSMC of the MESEMS trial**

Nicola De Stefano: MRI expert, University of Siena, Italy

Alan. Tyndall: Stem-cell therapy expert, University of Basel, Switzerland

Jeffrey. Cohen: Neurologist specialized in MS with experience in clinical trial to assess MSC, Cleveland Clinic Foundation, Cleveland, OH, USA

Cris Constantinescu: Neurologist with expertise in neuroimmunology related to MS, University of Nottingham, UK
